# Reutilisation of Water Contaminated by Mining Waste for the Encapsulation of Potentially Toxic Elements

**DOI:** 10.3390/ma15031076

**Published:** 2022-01-29

**Authors:** Jorge Suárez-Macías, Juan María Terrones-Saeta, Antonio Bernardo-Sánchez, Almudena Ortiz-Marqués, Ana Maria Castañón, Francisco Antonio Corpas-Iglesias

**Affiliations:** 1 Research Group TEP 222 “Materials and Mining Engineering”, Higher Polytechnic School of Linares, University of Jaen, 23700 Linares, Spain; facorpas@ujaen.es; 2 Department of Mining, Mechanical, Energetic and Civil Engineering, University of Huelva, 21819 Huelva, Spain; terrones@dimme.uhu.es; 3 Department of Mining, Topography and Structures, University of León (ESTIM), Campus de Vegazana s/n, 24071 Leon, Spain; antonio.bernardo@unileon.es (A.B.-S.); aortm@unileon.es (A.O.-M.); amcasg@unileon.es (A.M.C.)

**Keywords:** encapsulation of potentially toxic elements, potentially toxic elements, mining-contaminated water, mining waste, ceramics, circular economy, sustainability, construction materials

## Abstract

Mining activities are essential for a population’s development; however, they also produce negative effects such as the production of waste, an impact on flora and water pollution. On the other hand, construction is one of the sectors which is most demanding of raw materials, with one of the main such materials being water. For this reason, this research evaluates the feasibility of incorporating water contaminated by mining waste into ceramic materials for bricks. In this way, the use of water is reduced and, on the other hand, the contaminating elements of the mining water are encapsulated in the ceramic matrix. To achieve this, the clay used and the contaminated water were first analysed, then different families of samples were conformed with different percentages of contaminated water. These samples were tested to determine their physical and mechanical properties. At the same time, leachate tests were carried out to determine that the ceramic material created did not cause environmental problems. The test results showed that the physical and mechanical properties of the ceramics were not influenced by the addition of contaminated water. On the other hand, the leachate tests showed that encapsulation of most of the potentially toxic elements occurred. However, the use of contaminated water as mixing water for ceramics could only be performed up to 60%, as higher percentages would leach impermissible arsenic concentrations. Accordingly, a new way of reusing water contaminated by mining activities is developed in this study, taking advantage of resources, avoiding environmental pollution and creating economic and environmentally friendly end products.

## 1. Introduction

Mining activity is essential in order to obtain the resources necessary for the large quantity of products consumed by the population [1,2]. This activity produces aggregates for construction materials [3], energy resources for energy production [4,5] and metallic resources [6] for countless industrial products. Consequently, the need for its development is essential for maintaining the quality of life of the world’s population. However, during its activity and development, this sector produces a significant environmental effect on the environment [7]. This environmental impact is mainly due to the alteration of the landscape [8], the influence on the territory [9], the emission of noise [10] and most importantly, the production of waste [11] and the impact on surface water and groundwater [12].

New environmental regulations governing mining activities are becoming increasingly restrictive [13], mainly due to the knowledge of previous cases of serious environmental damage and the increased environmental awareness of the population. Consequently, these new regulations regulate, quantify, control and reduce environmental affections. Nevertheless, the applicable environmental regulations are relatively new, so there are currently abandoned mining operations from decades ago whose waste is currently producing significant environmental affections [14].

As mentioned above, mining activity inevitably has an impact on surface and groundwater, as it causes diversions of the riverbed and even significant modifications of aquifers. In turn, this water can be contaminated with chemical elements that are alien to its composition, causing a major environmental problem. In particular, metallic mining, through which polymetallic sulphides are found, is the mining activity that produces the most water pollution [15]. This results in water in this type of exploitation being commonly found with high concentrations of metallic elements such as iron, copper, zinc and even arsenic, as well as a very varied pH [16]. Consequently, this water presents an environmental problem and must be treated [17].

For treatment of these waters, the processes that have been used take the form of chemical precipitation, i.e., raising the pH to basic pH with the addition of lime, usually to precipitate the metallic elements in solution, and then removing them by decantation [18]. Other authors have carried out research to concentrate and extract the most economical chemical elements by hydrometallurgical processes [19]. However, both processes involve an economic cost that sometimes makes their application impossible.

In particular, at the beginning of the 20th century, there was significant lead-mining activity in the mining district of Linares in southern Spain [20]. This intense mining activity focused on the extraction of lead sulphides, for subsequent concentration. Therefore, a considerable amount of waste of different particle sizes was produced, in which it was usual to find polymetallic sulphide ores. This waste, since it belongs to old mines where there were no environmental regulations applicable at that time, was in most cases deposited in the open without any kind of control. As a result, contact from this waste with rainwater or small river beds creates water pollution, mainly with metallic elements [21]. However, because part of the mineralisation in the Linares mining district contains a significant amount of carbonate rocks, the pH of this type of water is not acidic [22]. Therefore, the presence of metallic elements such as zinc, copper, etc., is very low, and their extraction by hydrometallurgical techniques is not of interest. However, other elements such as arsenic are found in solution in the water up to pH levels much higher than those of the metallic elements detailed above, which is a highly polluting element for flora and fauna and is difficult to treat [23].

Parallel to this, in the vicinity of the mining district of Linares there are large companies dedicated to the extraction of clay and the manufacture of ceramic materials. It should be noted that the construction sector is one of the most polluting sectors in existence today [24]. This is mainly due to the enormous quantities of raw materials consumed in the manufacture of the products necessary for this sector. Consequently, and in order to alleviate this negative effect, various research projects have been carried out in which waste is used as raw material for the manufacture of these construction products. These include the use of biomass ash [25], mining waste [26], diatomaceous earth [27], organic waste [28], etc., for ceramic materials. However, in the process of conforming ceramic materials there is also a significant demand for water, approximately 20% per tonne of the final product [29]. Water is subsequently lost as water vapour during the sintering process and is removed from the material in the form of water vapour.

In view of the above, contaminated water from the mining district of Linares is used in this study for the conforming and production of ceramic materials. These mining waters, containing very high concentrations of arsenic, are used in different percentages of the total water required for the mixing process, determining the feasibility of incorporating the same for ceramics manufacture, as well as the retention of the arsenic element in the material produced. In this way, contaminated mining water is reused without the need to use natural water, and the contamination produced by this water is avoided through retention of the contaminating elements in the ceramic matrix.

To this end, this study first analysed and physically and chemically characterised the clay commonly used for the manufacture of ceramics, as well as the contaminated mine water. Subsequently, several families of samples were conformed with 20% mixing water to the material. Each family had a different percentage of contaminated mine water, from 0% to 100%. With the families of samples conformed, the sintering process was developed, subsequently evaluating the physical and mechanical properties of the different samples. At the same time, and with the final objective of retaining the contaminating elements of the mine water in the ceramic matrix (essentially arsenic), leaching tests were carried out to corroborate the viability of the process. 

The conclusions of the research showed that using mine water for the mixing of ceramic products produced negligible variations in their physical and mechanical properties. This in turn showed that it was possible to incorporate a high percentage of mine water in the mixing process without subsequent problems element contaminant leaching.

## 2. Materials and Methods

In this section, the materials used in this research and the tests carried out to corroborate this initial hypothesis are described. This initial hypothesis consists of determining the viability of using mining water with potentially toxic elements for the conformation of ceramic materials destined for bricks. In this manner, the contamination produced by the toxic elements in the water is avoided and retained in the ceramic matrix.

### 2.1. Materials

The materials used in this study correspond directly to the usual materials used in the manufacture of ceramic materials, i.e., clay as the principal material and water as the auxiliary element for conforming different ceramic pieces. However, this water, as mentioned above, will be partially or totally substituted by contaminated mining water, subsequently evaluating the maximum percentage of mining water that can be added to the process without producing considerable changes in the physical and mechanical properties of the conformed ceramic material. At the same time, it has been considered that the leaching of ceramics conformed with mining water does not produce contaminating leachates with concentrations of harmful chemical elements higher than those established by the regulations.

In the following sections, the materials used in the research—clay, distilled water and water contaminated by mining activities—are described in more detail.

#### 2.1.1. Clay

The clay used belongs to mining operations near the mining district of Linares, Spain. The geological diversity of the area, in the southern part of Spain, allows the development of highly fractured granites in which different polymetallic sulphides (mainly lead sulphides) are deposited by hydrothermal processes, as well as clay materials, which have created a very important ceramic industry at a national and European level, destined to the manufacture of construction materials, mainly bricks.

Ceramic industry in the indicated area mainly develops bricks with red clay, this being the same material used in this study. This clay presents optimal qualities for its use in detailed purpose, later showing the tests carried out to verify these characteristics.

It should be noted that the sample was taken in an unaltered form, taking several representative samples of the total exploitation. It should also be mentioned that this clay was dried at 105 ± 5 °C then crushed and sieved through a 0.25 mm sieve in order to develop a process similar to that carried out in industry. The sample thus prepared was used throughout the methodology, both for characterisation tests and for ceramic conforming.

It is important to note that moisture is not detrimental to the conforming process; it should simply be taken into account during the production process in order to avoid an excess of mixing water which could damage the quality of the material. However, in this study, this variable is eliminated in order not to affect superfluous variables that do not provide any information and that obscure the quality of the research.

#### 2.1.2. Water Contaminated by Mining Activities

**Figure 1:**
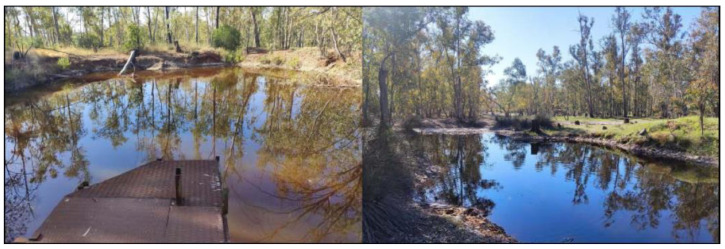
Image of the contaminated water reservoir.

The contaminated water belongs to the Linares mining district, Figure 1. This water has accumulated in old mining ponds used in the past for the concentration and treatment of the minerals extracted. Currently, the water accumulates as a result of surface runoff phenomena, depositing the water in these lower-elevation ponds. The water pollution that accumulates in these mining ponds seems to correspond to the leaching of potentially toxic elements from mining waste deposited at higher levels without any kind of environmental protection. Rainfall periods bring the water into contact with this mining waste and then leach out the potentially toxic elements. This contaminated water travels superficially until it accumulates in the detailed ponds at lower altitudes.

It is usual, therefore, for the concentration of toxic elements in contaminated water to vary according to rainfall periods. Therefore, samples were taken from the different ponds over the course of a year and analysed. It was found that the water in the different ponds was very similar; however, the highest concentration of potentially toxic elements in the water occurred during periods of low rainfall in all cases. This is because evaporation phenomena—as well as the scarcity of rain—cause the water level in the ponds to decrease, resulting in a higher concentration of the chemical element detailed. The water corresponding to this period was used for the conforming of ceramic materials, as it corresponds to the most unfavourable case.

Water contaminated by mining waste was mixed in different percentages with distilled water. The properties of the distilled water used are shown in Table 1.

### 2.2. Methodology

Methodology used in this research is based on a series of logically ordered tests that make it possible to assess the viability of using water contaminated by mining activities as mixing water in ceramic materials. To this end, the tests are divided into three large blocks according to the purpose for which they are to be carried out. Firstly, the raw materials are characterised, these being the contaminated water and the clay used. Subsequently, different families of samples are conformed with different percentages of contaminated water, and physical and mechanical tests are carried out to determine the variability. Finally, leachate tests are executed to determine whether the potentially toxic elements present in the contaminated water are retained in the ceramic matrix, determining the maximum percentage of contaminated water susceptible to be used, producing a quality ceramic and without environmental hazard. In the following sections, details of the tests for each section are described in depth.

#### 2.2.1. Raw Material Characterisation

Firstly, tests were carried out to characterise the materials used in the research. These raw materials, as mentioned above, are clay and water contaminated by mining waste.

Clay was characterised in order to determine its suitability for the conforming of ceramic materials. Initially, its physical properties were determined, through the calculation of the particle density of the clay by means of the pyknometer method, standard UNE-EN 1097-7 [30]. In turn, the liquid limit, plastic limit and plasticity index of the clay were determined, according to the UNE-EN ISO 17892-12 [31] standard, in order to determine the quality of the clay for use in ceramic materials.

Once the physical properties of the clay had been determined, the chemical characterisation was carried out. For this, the elemental analysis test was carried out, quantifying the percentage of carbon, hydrogen and nitrogen existing in the samples. This test analyses the gases produced during the calcination of the sample at a temperature of 950 ± 5 °C, identifying and quantifying the aforementioned chemical elements of lower atomic weight. The elemental analysis test was performed with LECO’s TruSpec Micro equipment (TruSpec Micro, LECO, St. Joseph, MI, USA). In turn, the loss on ignition was evaluated at a temperature of 950 ± 5 °C, determining the mass variation that occurs in the sample before and after subjecting it to the detailed temperature. Finally, in order to identify and quantify the higher atomic weight elements present in the clay, the X-ray fluorescence test was performed with commercial equipment (ADVANT’XP+, Thermo Fisher, Waltham, MA, USA).

On the other hand, the water contaminated by mining waste was taken directly from the pond in which it was accumulated in order to obtain the samples. These samples were chemically characterised by inductively coupled plasma mass spectrometry (7900, Agilent, Santa Clara, CA, USA). In this form, it was possible to know the chemical composition of the contaminated water, as well as the potentially toxic elements existing in the water and the concentration in which they were found.

#### 2.2.2. Conforming of Ceramic Samples—Physical and Mechanical Tests

After characterising the clay and the contaminated water, different families of samples were conformed with both. The different families of samples were manufactured with 20% mixing water on clay mass, i.e., mixing water composed of different combinations of contaminated water and distilled water. In this way, it was possible to evaluate the variation produced in the physical and mechanical properties of the ceramics by the addition of contaminated water. The families of samples manufactured, as well as the percentage of contaminated water and distilled water in each of the families, are detailed in Table 2.

To determine which families of samples were to be made with clay and contaminated water, 12 samples were conformed for each family. The conforming process for all samples was similar, therefore obtaining, comparable results. This process consisted of mixing the clay with 20% mixing water, made up of the different percentages of contaminated water and distilled water corresponding to each family and detailed in Table 2. After this mixing process, the material was compacted to simulate the extrusion process that takes place in the industry. For this, the mixture of clay and water was poured into a 60 mm × 30 mm metal matrix and then a load of 30 MPa was applied. When the compaction process was finished, the samples were extracted and dried for 24 h at a temperature of 105 ± 2 °C and then subjected to the sintering process.

The sintering process consisted mainly of subjecting the samples to a temperature of 950 ± 5 °C for 1 h.

To achieve this temperature, a heating ramp of 4 °C per minute was used. Finally, the samples were removed from the furnace once they were at room temperature, and physical and mechanical tests were performed on all families of samples. It is important to note that the parameters of compaction pressure, percentage of water and temperature were set in order to obtain ceramics with similar characteristics to those obtained in industrial processes.

The conformed samples of all the families according to the described procedure were tested to determine their physical and mechanical properties, thus evaluating the variation of these properties due to the incorporation of the contaminated water. The first physical tests carried out were linear shrinkage and weight loss during the sintering process, according to the UNE-EN 772-16 standard [32]. These tests, which quantify the variation in the dimensions of the samples and the weight, respectively, before and after the sintering process, are essential to obtain a quality product with the commercial specifications required in ceramics, as the final product must have certain dimensions and weight.

Subsequently, capillary water absorption (standard UNE-EN 772-11) [33], cold water absorption (standard UNE-EN 772-21) [34] and determination of open porosity and bulk density (UNE-EN 772-4) [35] tests were carried out. These tests, carried out by hydrostatic methods, faithfully quantify the structure of the conformed ceramic, determining its quality, as well as the possible properties of resistance, thermal insulation, acoustic insulation, etc., that can be developed.

The last of the physical tests carried out was the determination of the colour of all families of ceramics. This property, which is not limited by regulations, is of paramount importance in the marketing of the materials, as manufacturing companies want the colour of the ceramics to remain unchanged by the addition of the waste. Consequently, this test was carried out in order to determine that the addition of contaminated water did not produce substantial variations in the colour of the ceramic. For this purpose, a so-called colourimeter was used, more specifically the PCE-RGB 2 (RGB-2, PCE, Meschede, Germany).

Finally, once the main physical properties of the ceramic families had been determined, the mechanical strength of all the samples was determined according to the UNE-EN 772-1 standard [36]. This test is essential, since the element for which the ceramic material is intended is bricks, and according to the standard, it must have a compressive strength of over 10 MPa. In addition, the variation in strength due to the addition of contaminated water is desirable to be as small as possible.

#### 2.2.3. Leaching Tests

Lastly, to evaluate the retention of potentially toxic elements of the contaminated water in the ceramic matrix and, consequently, the toxicity of the different families of ceramics and their potential to leach contaminants, the TCLP test [37] was performed on all families of ceramics.

For this purpose, the ceramic was ground to obtain samples with a particle size of less than 10 mm. Then, the leaching fluid was made according to the Environmental Protection Agency (EPA) standard [37], being composed of glacial acetic acid (5.7 mL) and 64.3 mL of sodium hydroxide solution (1N) diluted in 1 L of distilled water. The proportion of leached fluid was 20 times the mass of the sample. The mixture of the sample and the prepared solution was kept stirred for 18 ± 2 h at a temperature of 22 ± 3 °C. Subsequently, the solution was filtered through a glass fibre filter with an effective pore size of 0.7 micrometres and acidified with nitric acid to a pH of 2. The extracted liquid was analysed by inductively coupled plasma mass spectrometry (7900, Agilent, Santa Clara, CA, USA) to determine the concentration in the obtained leachate of the potentially toxic elements regulated by the EPA [37] for this type of material. The concentration limits set by the EPA [37] are shown in Table 3.

Consequently, this test limits the maximum percentage of contaminated water that can be used as mixing water for ceramics, and in turn assesses whether the potentially toxic elements of the contaminated water are properly retained in the ceramic matrix.

## 3. Results and Discussion

Results and discussion of the tests appear in this section in the same order in which they have been presented in the methodology. These results allow us to obtain a series of conclusions that will condition the final hypothesis, which is the verification of the viability of using water contaminated by mining waste as mixing water for ceramic conforming.

### 3.1. Raw Material Characterisation

The characterisation of the clay showed that this material had a particle density of 2.44 g/cm^3^. This density is usual in the different types of clays analysed by various authors, being ideal for use in ceramic materials.

In turn, the liquid and plastic limits of the clay used, as well as its plasticity index, are shown in Table 4.

As can be seen in Table 4, the clay used has a high plasticity. Consequently, this result conditions an excellent quality of the material for ceramic conforming, as it demonstrates the small particle size of the clay, as well as its ease in conforming with water.

Determining the principal physical characteristics of the clay, the chemical composition of the clay was assessed. The first of the tests carried out was elemental analysis, showing the percentage of the chemical elements carbon, nitrogen and hydrogen in the sample. The results of the elemental analysis test are shown in Table 5.

The results of the elemental analysis test of the clay used in this research for ceramic conforming, detailed in Table 5, reflect a mainly inorganic composition of the material. This is due to the low percentage of carbon and hydrogen in the sample. In other words, the percentage of organic matter in the clay is practically nonexistent. In turn, the low percentage of carbon also demonstrates the low proportion of carbonates in the clay, which is to be expected considering that the clay is composed of hydrated aluminium silicates. However, a high percentage of organic matter or carbonates in a clay would reflect the low quality of the product, as it could correspond to an impure material contaminated by external agents. This is not the case of the analysis, and the results obtained from the elemental analysis test are favourable for the use of the evaluated clay in ceramics.

On the other hand, the loss on ignition test shows, as expected from the data analysed above, that the clay has a low proportion of volatile elements, carbonates, organic matter and other components of lower atomic weight. This is due to the fact that the variation in mass due to loss on ignition of the clay is 7.90 ± 0.35%.

With the aim of determining and quantifying the higher atomic weight elements present in the clay, an X-ray fluorescence test was conducted, showing the results presented in Table 6.

The X-ray fluorescence test confirmed the chemical composition of a quality clay. As mentioned above, this is because the clay consists of hydrated aluminium silicates, so its main chemical composition is silicon and aluminium. It is worth highlighting the presence of a considerable percentage of iron; this element is beneficial for the conforming of ceramic materials, as it provides the material with adequate resistance. The same is true of magnesium. On the other hand, calcium is found in low proportion, which is beneficial if it is considered that calcium carbonates can damage the conformed ceramic, developing inadequate physical and mechanical characteristics. The other chemical elements are in low proportions, so their influence on the ceramic material conformed with the detailed clay will be reduced. It should be noted, however, that there are no potentially toxic elements such as chromium, lead, arsenic, cadmium or barium in the chemical composition of the clay.

Consequently, the results of the physical and chemical characterisation of the clay show that it is a quality material, ideal for conforming ceramic materials for bricks.

On the other hand, the water contaminated by mining waste was characterised. The water presented a significant turbidity, with a brownish colour and an intense and highly unpleasant odour. This water was analysed by inductively coupled plasma mass spectrometry, with the results shown in Table 7.

Table 7 shows the chemical elements found in solution in the water contaminated by mining waste, as well as the concentration in which they are found. Results principally show that the water is of low-quality, derived mainly from polluting industrial processes such as mining, as the chemical elements boron, sodium, silicon and sulphur are found in high proportion. Furthermore, it should be taken into account that the concentration of arsenic in water for human consumption is limited to 10 ppb and this water has 452.205 ppb of arsenic. Therefore, it can be stated that the concentration of arsenic is very high and that this water can cause major environmental problems if it is not treated. This arsenic concentration derives, as expected, from the leachate from mining waste. These wastes, as mentioned above, belong to lead sulphide mines where polymetallic sulphides, such as arsenic sulphide, are commonly found. However, it can be observed that other chemical elements such as copper, zinc, iron, nickel, titanium, etc., are found in low proportions, although they are easily found in mining waste. This is because the Linares mining district does not produce acid mine water, since there are carbonate rocks. Therefore, the acidity of the water is reduced and does not keep the chemical elements detailed above in suspension, unlike arsenic. At the same time, it should be noted that the chemical elements controlled by the EPA [37] for construction materials are chromium, lead, arsenic, barium and cadmium, as this regulation considers them potentially toxic and limits their concentration in the leachate produced from the ceramic. However, these chemical elements are practically nonexistent in the clay and are very insignificant in the contaminated water (with the exception of arsenic, which is found in high proportions). Therefore, the ceramics conformed with the contaminated water must be leached and evaluated to determine that the concentration of arsenic in the leachate is lower than that set by the above-mentioned regulation. Otherwise, the maximum percentage of contaminated water that can be incorporated without causing environmental damage from the production of the ceramic material will be calculated.

### 3.2. Conforming of Ceramic Samples—Physical and Mechanical Tests

After the characterisation of the clay and water contaminated by mining waste, checking its environmental hazard, the different families of samples were conformed. These families of samples were composed of clay and 20% of the water from mixing the dry clay mass. Each family had different percentages of contaminated water and distilled water, from 0% contaminated water to 100%, always referring to the percentage of mixing water. In this way, it was possible to evaluate the variation of the physical and chemical properties produced in the ceramic by using the mixing water in increasing percentages. 

**Figure 2:**
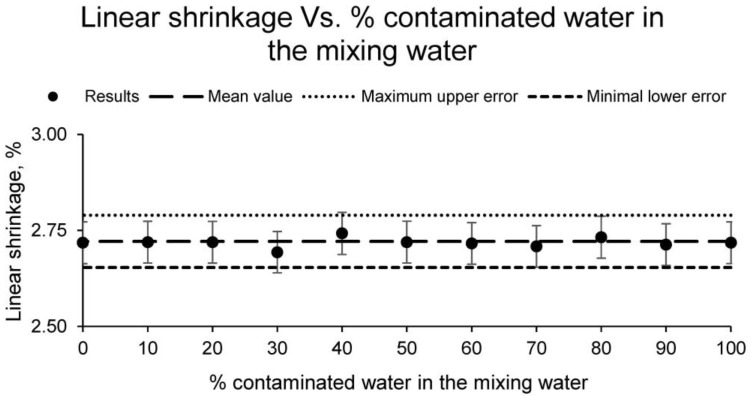
Linear shrinkage of the families of ceramics conformed with different percentages of contaminated water and distilled water as mixing water (20% on clay mass).

The first of the tests carried out to determine the physical properties of the different families of ceramic samples was the linear shrinkage test. The results of this test are shown in Figure 2.

The results of the linear shrinkage, after the sintering process, of the different families of samples conformed with clay and contaminated water show similarities. In other words, the variation in linear shrinkage caused by using water contaminated by mining waste is null, since the values obtained for all the families differ by less than 5%. Consequently, it can be stated that the use of water contaminated by mining waste as mixing water does not alter this physical property. 

**Figure 3:**
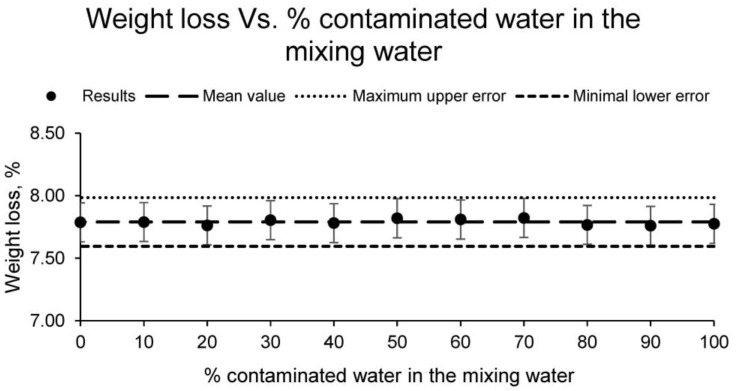
Weight loss of the families of ceramics conformed with different percentages of contaminated water and distilled water as mixing water (20% on clay mass).

On the other hand, the weight loss that occurs in the sintering process was calculated for the different families of samples. The results of this test are shown in Figure 3.

The weight loss for all families of samples, as with linear shrinkage, does not differ by using contaminated water as the mixing water, as the values obtained differ by less than 5%. Therefore, the conformed ceramic presents a weight loss during the sintering process of 7.79%, independently of the use of contaminated water or distilled water as mixing water. It should be noted that this physical property, together with the linear shrinkage, are essential properties that must be known about a ceramic. Since the final ceramic product must have a specific size and weight, it is necessary to know these properties beforehand in order to use the correct material and mould.

**Figure 4:**
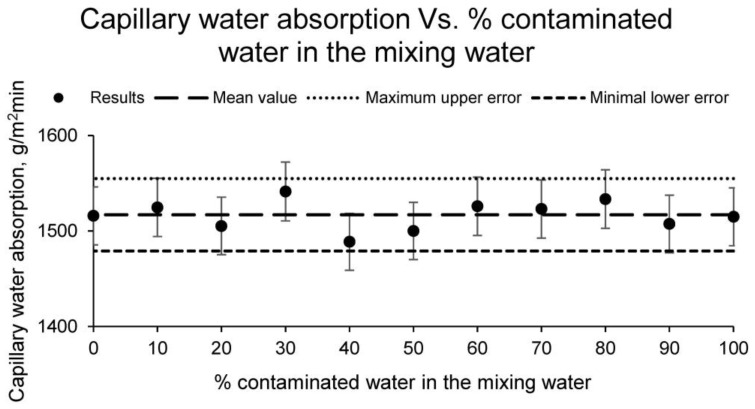
Capillary water absorption of the families of ceramics conformed with different percentages of contaminated water and distilled water as mixing water (20% on clay mass).

Subsequently, the capillary water absorption test was carried out for all families of samples. The results of this test are shown in Figure 4.

Capillary water absorption is an essential physical property that indirectly determines the structure of the ceramic. A ceramic with a more open structure and a higher number of interconnected pores will reflect a higher capillary water absorption. On the contrary, a more compact, more resistant ceramic with a much more closed structure presents a lower water absorption by capillarity. In this case, all the families have obtained similar results of water absorption by capillarity, reflecting an average value of 1517 g/(m^2^ min). This value shows a quality ceramic which is resistant and has a structure with a reduced number of unconnected pores.

**Figure 5:**
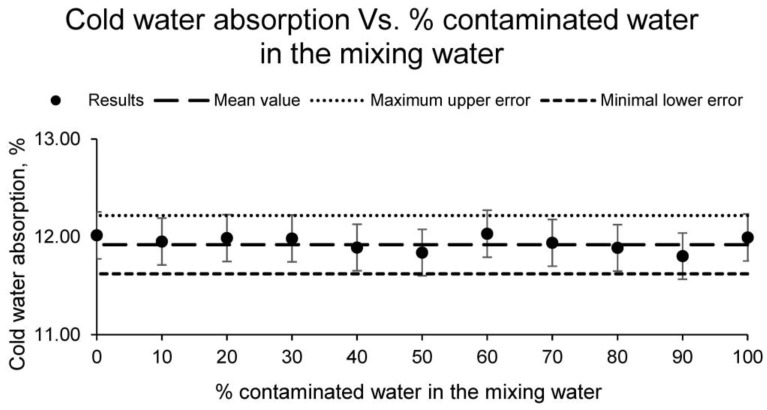
Cold water absorption of the families of ceramics conformed with different percentages of contaminated water and distilled water as mixing water (20% on clay mass).

Moreover, Figure 5 shows the cold water absorption of the different families of conformed ceramic samples.

As Figure 5 depicts, as in the previous cases, the cold water absorption of the different families of samples is independent of the percentage of contaminated water used as mixing water, showing an average value of 11.92% and a difference between the results of all the families of less than 5%. It should be noted that this property is very interesting, and essential to calculate, for all those ceramic materials that are outdoors or in direct contact with moisture. This is due to the fact that a ceramic tile with a higher absorption of cold water will absorb rainwater or humidity from the ground during its useful life, increasing its weight unnecessarily and unnecessarily overloading the structure on which it is supported. In this case, the obtained values are low, corroborating the results obtained in the previous tests, which show a quality ceramic with a closed structure.

**Figure 6:**
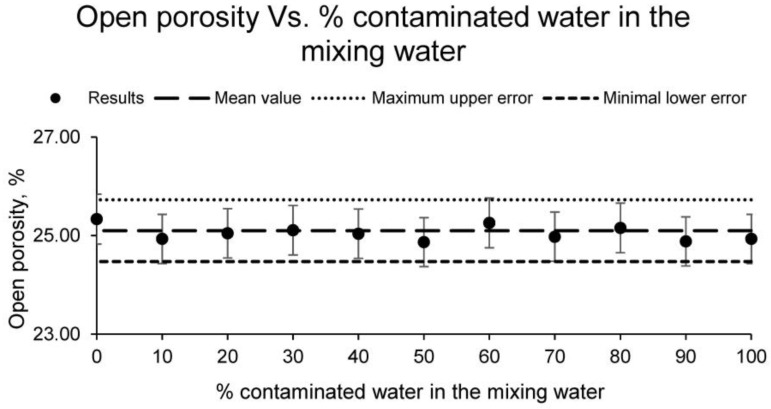
Open porosity of the families of ceramics conformed with different percentages of contaminated water and distilled water as mixing water (20% on clay mass).

In addition, the open porosity of the different families of ceramics was calculated, showing the results in Figure 6.

As with the previous parameters, the open porosity is similar for all families of samples. The results obtained for all families differ among themselves by less than 5%, obtaining an average open porosity value of 25.10%. These porosity results are adequate for ceramics intended for brick manufacture. However, it should be noted that higher porosity also leads to better thermal and acoustic insulation, since the higher the number of pores, the lower the conductivity. Therefore, in some types of ceramic materials, it is of interest that this porosity is higher.

**Figure 7:**
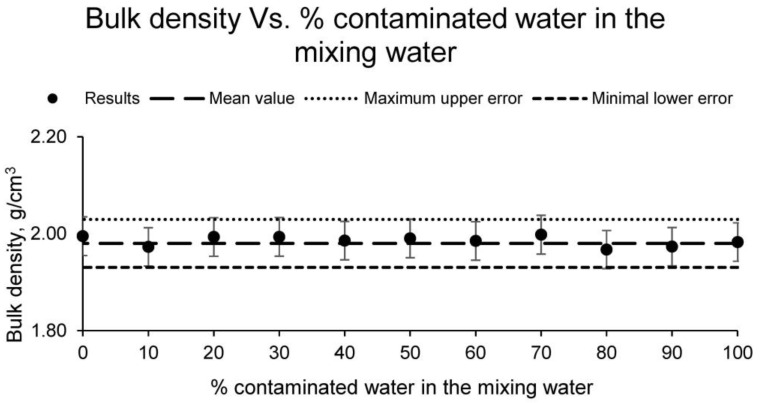
Bulk density of the families of ceramics conformed with different percentages of contaminated water and distilled water as mixing water (20% on clay mass).

Next, the bulk density of the different families of samples was calculated. The results of this test are shown in Figure 7.

Bulk density is an essential physical property for a ceramic, which indirectly determines other properties such as strength. A lower bulk density for the same material conditions a lower strength and a more open structure. In this case, all families of samples showed similar bulk density values, the results differing by less than 5% and reflecting an average value, usual for ceramics, of 1.98 t/m^3^.

Finally, the colour coordinates of all the conformed sample families were calculated. The test aimed to determine whether the addition of contaminated water produced variations in the colour of the ceramic, making it visually unattractive for the market. The results of the colourimetric test are shown in Table 8 for all the sample families.

The colour coordinates of the different families of ceramics show that the variations produced in the colour of the ceramics by using contaminated water as mixing water is totally insignificant.

**Figure 8:**
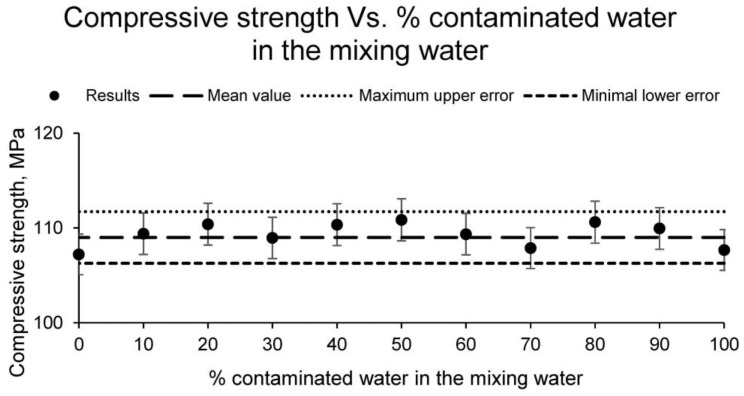
Simple compressive strength of the families of ceramics conformed with different percentages of contaminated water and distilled water as mixing water (20% on clay mass).

Consequently, the use of water contaminated by mining waste creates materials with physical properties similar to the traditional ones, without producing any variation. However, because the ceramics are intended for bricks, they must meet minimum mechanical characteristics. Therefore, the mechanical properties were evaluated by means of the compressive strength test, showing the values shown in Figure 8.

The compressive strength of the different families of ceramics, as with the physical properties calculated above, experiments no variation with respect to the use of water contaminated by mining waste. The compressive strength variations of the different families of conformed ceramics is less than 5%, showing an average strength value of 109 MPa. This value is an excellent result for a ceramic intended for the manufacture of bricks.

Consequently, and based on the results obtained, the use of water contaminated by mining waste as mixing water for ceramics does not affect the physical and mechanical properties of the conformed ceramics.

### 3.3. Leaching Tests

Chemical characterisation of the water contaminated by mining waste determined that it had potentially toxic elements, mainly arsenic. On the other hand, the physical and mechanical tests of the different families of samples conformed with clay and contaminated water as mixing water showed that the use of contaminated water to conform ceramics did not produce variations in physical and chemical properties. However, since contaminated water contains toxic elements, it should be verified that these elements are retained in the ceramic matrix. In turn, it must be verified that the leachates of the ceramic materials for construction have concentrations of potentially toxic elements lower than those established by the EPA regulations [37].

Therefore, leachate tests were carried out, as detailed in the methodology, in order to determine the maximum percentage of contaminated water that could be used as mixing water without creating environmentally harmful ceramic materials.

The chemical elements regulated by the regulations in this respect are detailed in Table 3. Chromium is limited to 5000 ppb; however, because the contaminated water has a very low percentage of chromium, all the families of ceramics conformed showed zero values of this element in their leachates. The same was true for cadmium, which is also found in low proportions in the contaminated water.

**Figure 9:**
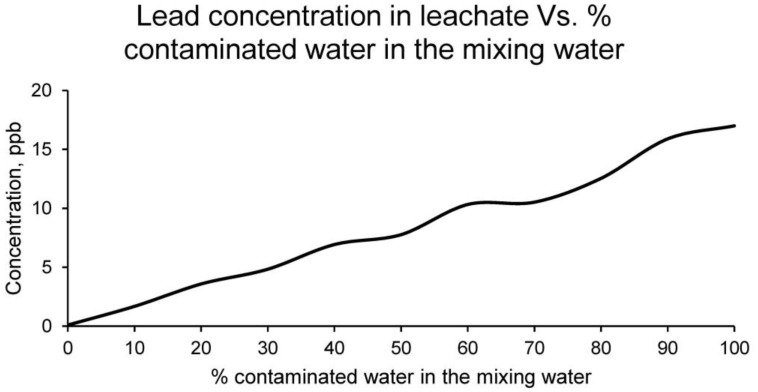
Lead concentration in leachates of the families of ceramics conformed with different percentages of contaminated water and distilled water as mixing water (20% on clay mass).

Lead, although in low proportion in the contaminated water, was found in the leachate of the different families of samples, reflecting the results shown in Figure 9.

It can be observed that the concentration of lead in the leachate is much lower than in the contaminated water, so it could be assumed that the ceramic matrix has retained part of this element. It should be noted that the higher the percentage of contaminated water as mixing water, the higher the concentration of lead in the leachate. However, in all families of samples, the concentration of lead is lower than the limit set by the regulations for this type of material. 

**Figure 10:**
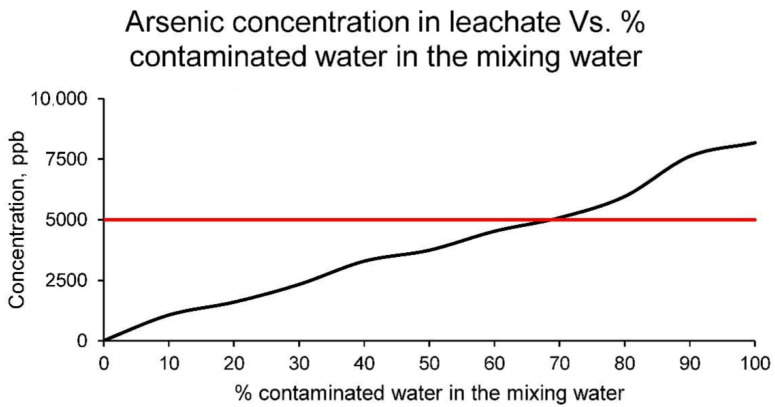
Arsenic concentration in leachates of the families of ceramics conformed with different percentages of contaminated water and distilled water as mixing water (20% on clay mass).

On the other side, the concentration of arsenic in the leachate of the different families of samples can be seen in Figure 10.

The arsenic concentration in the leachate of all sample families is much lower than the arsenic concentration in the contaminated water. Consequently, it can be stated that arsenic is largely retained in the ceramic matrix. However, the higher the percentage of contaminated water in the ceramic, the higher the concentration of arsenic in the leachate, even exceeding the EPA limits [37]. The families of ceramics that have arsenic concentrations in the leachate higher than the set limit are those with percentages higher than 60% of contaminated water as mixing water (20% of dry clay mass). Therefore, and because these families of ceramics may pose an environmental and health problem, the maximum percentage of contaminated water use is limited to 60% of the mixing water.

**Figure 11:**
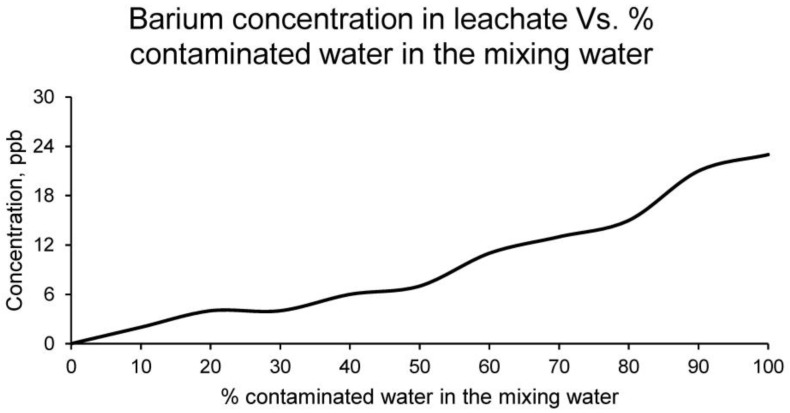
Barium concentration in leachates of the families of ceramics conformed with different percentages of contaminated water and distilled water as mixing water (20% on clay mass).

Ultimately, the concentration of barium in the leachates from the different families of ceramic samples is shown in Figure 11.

Barium has a very low concentration in the contaminated water, and consequently in the leachates of the families of ceramics conformed, not being higher than the limits set by the EPA [37]. Therefore, this chemical element is not a limiting factor for setting the maximum percentage of contaminated water that the ceramic can incorporate.

Consequently, and according to the results of the leachate tests, it can be stated that the detailed chemical elements existing in the contaminated water are retained in the ceramic matrix to a high proportion. However, the maximum percentage of contaminated water that can be used as mixing water is 60%, which would correspond to 12% of contaminated water on dry clay mass.

## 4. Conclusions

Results from the tests as described in the methodology permit a series of partial conclusions to be drawn that lead to the final conclusion. Therefore, the main conclusions that have been drawn from this research are detailed below.

- The clay used for ceramic conforming has an ideal density and plasticity for the conforming of ceramic materials. The chemical composition of this clay shows a usual composition of hydrated aluminium silicates, reflecting a reduced percentage of organic matter, carbonates, nitrogen and other volatile elements.
- Water contaminated by mining waste has a very low-quality chemical composition, with high concentrations of sulphur and sodium (among other elements) and a variety of potentially toxic elements. The concentration of arsenic in the contaminated water is remarkable, as it has a high proportion of this element and is a major environmental problem.
- Ceramics conformed with contaminated water as mixing water have the same physical properties as ceramics conformed with clay and distilled water. Thus, using the use of contaminated water in ceramic conforming does not influence the physical properties.
- The compressive strength of all families of samples conformed with contaminated water or distilled water is similar, and the use of contaminated water as mixing water does not influence the compressive strength of the ceramics.
- The leachates of the sample families conformed showed that arsenic, a potentially toxic element with a high proportion in the contaminated water, could be largely retained in the ceramic matrix. However, only the samples with less than 60% of contaminated water as mixing water had arsenic concentrations in their leachates below the concentrations set by the EPA [37].

As a consequence, it is possible to assert that ceramic bricks for construction can be manufactured with water contaminated by mining waste as a substitute for 60% of the mixing water, creating a resistant material with physical properties similar to traditional ceramics. This study is, therefore, an example of new actions that can be performed with mining water: first, avoiding the contamination produced by this water in the environment where it is found; second, to encapsulate the potentially toxic elements of the water in the ceramic matrix; and finally, to obtain a product of lower economic cost, which is safe for health and environmentally friendly.

## Figures and Tables

**Table 1:** Properties of the distilled water used.

Characteristic	Result
Appearance	Colorless liquid
Density at 20 °C	0.995 g/cm^3^
pH	7
Conductivity	2.5 μS/cm
Silica percentage	0.003 mg/L
Hardness	0.3 mg CaCO_3_/L

**Table 2:** Families of conformed ceramics with different percentages of contaminated water as mixing water.

Family	Contaminated Water, %	Distilled Water, %
C0	0	100
C1	10	90
C2	20	80
C3	30	70
C4	40	60
C5	50	50
C6	60	40
C7	70	30
C8	80	20
C9	90	10
C10	100	0

**Table 3:** Maximum concentrations of metals or toxic elements in the leachate according to the TCLP method (U.S. EPA) [37].

Metals	Maximum Allowable Concentration in the Leachate, ppb
Cr	5000
Pb	5000
As	5000
Cd	1000
Ba	100,000

**Table 4:** Liquid limit, plastic limit and plasticity index for the clay used.

Test	Value,%
Liquid Limit	38.5 ± 1.7
Plastic Limit	22.1 ± 1.0
Plasticity Index	16.4 ± 0.8

**Table 5:** Elemental analysis carbon, hydrogen and nitrogen for clay.

Sample	Nitrogen,%	Carbon,%	Hydrogen,%
Clay	0.04 ± 0.00	1.16 ± 0.05	0.65 ± 0.02

**Table 6:** Determination of the elemental composition of clay by X-ray fluorescence method.

Compound	Clay, WT%
SiO_2_	52.62 ± 0.25
Al_2_O_3_	17.83 ± 0.19
Fe_2_O_3_	7.84 ± 0.13
K_2_O	5.63 ± 0.12
MgO	3.44 ± 0.09
CaO	3.19 ± 0.09
TiO_2_	0.769 ± 0.038
Na_2_O	0.165 ± 0.015
P_2_O_5_	0.154 ± 0.008
MnO	0.154 ± 0.008
ZrO_2_	0.0379 ± 0.0049
V_2_O_5_	0.0357 ± 0.0031
SrO	0.0344 ± 0.0036
RuO_4_	0.0318 ± 0.0021
Rb_2_O	0.0273 ± 0.0048
PdO	0.0273 ± 0.0040
S	0.0247 ± 0.0013
NiO	0.0233 ± 0.0020
PtO_2_	0.0184 ± 0.0039
Cr_2_O_3_	0.0164 ± 0.0023
Cl	0.0095 ± 0.0008
Co_3_O_4_	0.0078 ± 0.0023
MoO_3_	0.0063 ± 0.0018
ZnO	0.0062 ± 0.0027

**Table 7:** ICP-MS of the water contaminated.

Element	Concentration, ppm
B	2.871
Na	5444.431
Mg	58.361
Al	0.109
Si	1228.301
P	1.010
S	2699.736
K	313.321
Ca	18.961
Ti	0.024
V	0.015
Cr	0.001
Mn	0.062
Fe	0.294
Co	0.001
Ni	0.001
Cu	0.039
Zn	0.046
As	452.205
Se	0.379
Mo	0.289
Cd	0.001
Sb	0.978
Ba	0.142
Pb	0.098

**Table 8:** Colour coordinates (red, green and blue) of the families of ceramics conformed with different percentages of contaminated water and distilled water as mixing water (20% on clay mass).

Family	Red	Green	Blue
C0	280	163	125
C1	276	167	125
C2	278	165	122
C3	282	164	125
C4	278	165	127
C5	282	164	126
C6	285	161	127
C7	279	161	123
C8	274	164	128
C9	280	167	127
C10	278	166	122

## Data Availability

Data is contained within the article.
